# Multipolar Mapping for Ventricular Tachycardia Ablation in a Patient with Left Ventricular Assist Device

**DOI:** 10.19102/icrm.2021.120123S

**Published:** 2021-01-15

**Authors:** Paolo Domenico Dallaglio, JuliÁn Rodríguez Garcia, Marta Aceña, Andrea Di Marco, Valentina Faga, Laia Garrido, Laia Llorca, Ignasi Anguera

**Affiliations:** ^1^Arrhythmias Unit, Heart Disease Institute, Bellvitge University Hospital, Barcelona, Spain; ^2^Abbott, Chicago, IL, USA

**Keywords:** HD grid mapping catheter, hemodynamic support, left ventricular assist device, multipolar mapping, ventricular tachycardia ablation

Left ventricular (LV) assist devices (LVADs) are a treatment option in patients with advanced heart disease. Ventricular tachycardias (VTs) are common in patients with continuous-flow LVADs and have been associated with increased mortality rates.^1^ In this new clinical scenario, ablation has been proposed as a very promising treatment tool.^2^ Herein, we report a case of VT ablation in a 67-year-old patient with ischemic cardiomyopathy and severe LV dysfunction who had previously received an LVAD (HeartMate III) implanted as a bridge to heart transplantation. The patient was admitted to the intensive care unit due to multiple VT episodes with a suspected inferolateral and apical origin **(Figure 1)**. VT episodes were resistant to antiarrhythmic drugs and to antitachycardia pacing, so a decision was made to perform VT ablation.

After a single transseptal puncture, a steerable sheath (Agilis®) was placed in the LV and a multipolar mapping catheter (Advisor™ HD Grid Mapping Catheter, Sensor Enabled™) was advanced until the zone of interest was reached. Special attention was needed to avoid contact between the catheter tip and the inflow cannula of the LVAD, which was placed in the apex^3^ **(Figure 2)**. The Advisor™ HD Grid catheter is characterized by significant tip flexibility and adaptability to the cavity surface, which allow the operator to conduct very detailed anatomical mapping of the LV and high-definition reconstructions of the area surrounding the inflow cannula that projects into the LV cavity **(Figure 3)**.

Voltage and activation mapping were performed, showing late and highly fractionated potentials all around the cannula, especially in the inferoapical–septal aspect **(Figures 4 and 5)**. Programmed ventricular stimulation induced three different VTs, including two corresponding with clinical ones; unfortunately, all induced VTs were nonsustained and complete activation mapping was not feasible. Nevertheless, multipoint rapid acquisition and high-definition mapping with the Advisor™ HD Grid catheter allowed us to localize areas of putative critical isthmus. These areas, located in the inferior–apical–septal aspect of the inflow cannula border zone, showed very slow conduction and mid-diastolic electrograms during nonsustained VTs **(Figures 6 and 7 and Video 1)**. Ablation was performed with the irrigated TactiCath™ SE catheter, targeting areas of previously annotated late potentials and mid-diastolic electrograms, especially in the surrounding of the inflow cannula. The postablation programmed ventricular stimulation did not induce any VT. Final remapping showed complete abolition of all late potentials **(Figure 8)**.

## Figures and Tables

**Figure 1:**
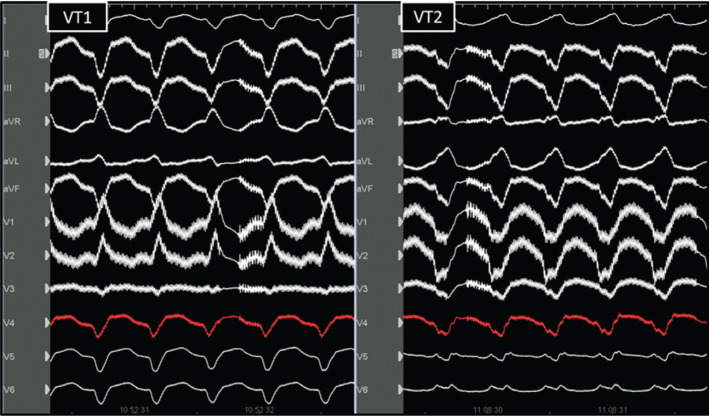
A 12-lead tachycardia electrocardiogram.

**Figure 2:**
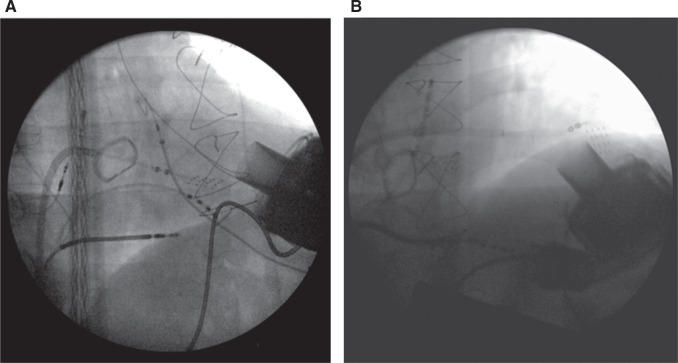
Right anterior oblique fluoroscopy image of two positions of the Advisor™ HD Grid catheter during mapping around the inflow cannula.

**Figure 3:**
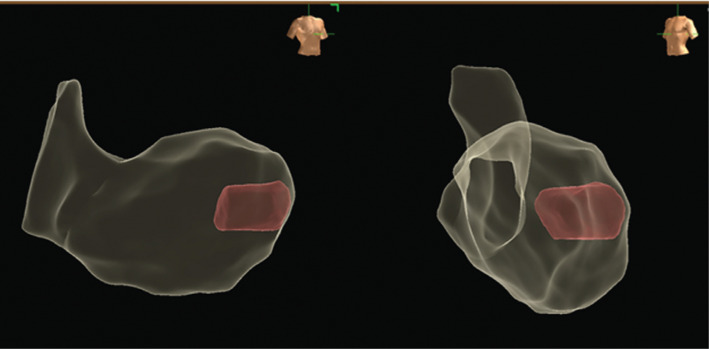
Left and right anterior oblique projections of the anatomical reconstruction showing the inflow cannula of the LVAD (red transparency).

**Figure 4:**
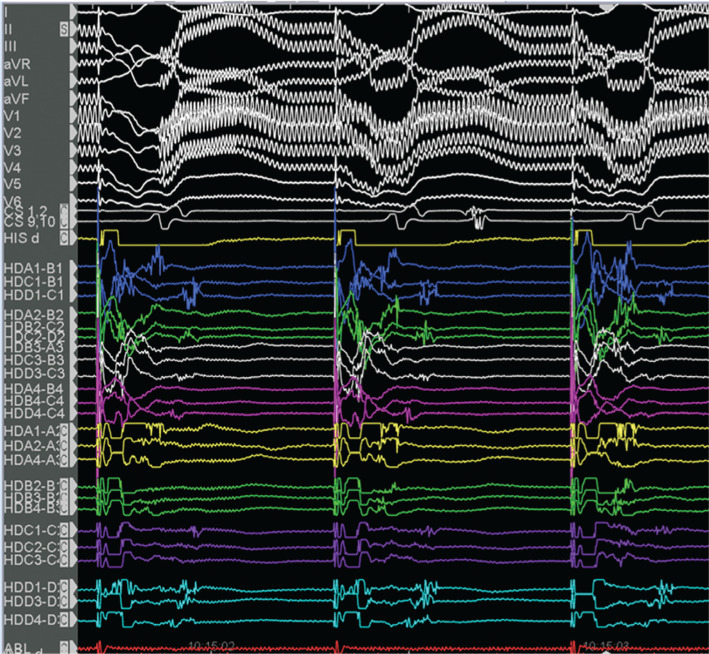
Late and highly fractionated potentials mapped with the Advisor™ HD Grid catheter.

**Figure 5:**
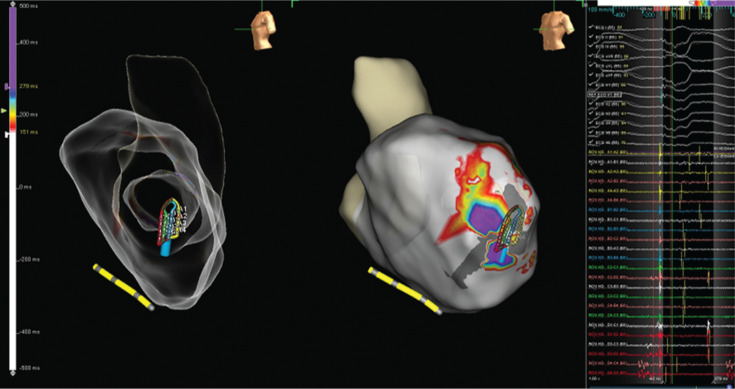
Late-potential activation map showing an area of late activation around the inflow cannula.

**Figure 6:**
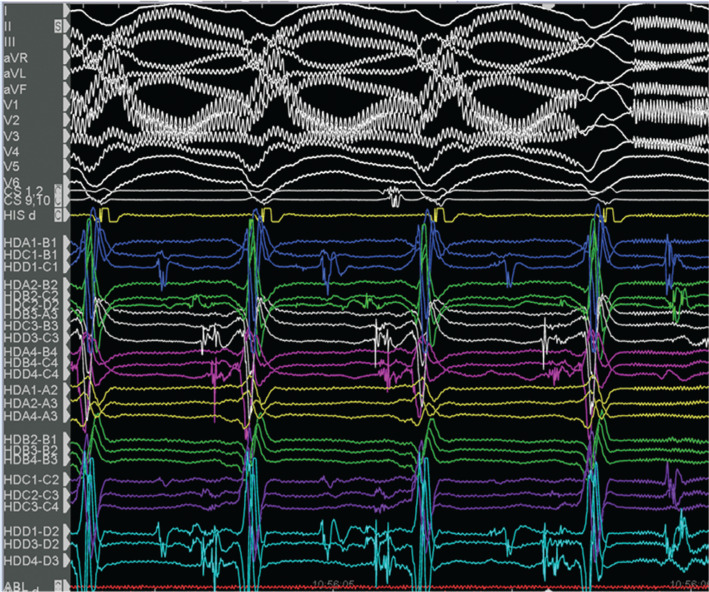
Mid-diastolic electrogram during VT1 mapped with the Advisor™ HD Grid catheter.

**Figure 7:**
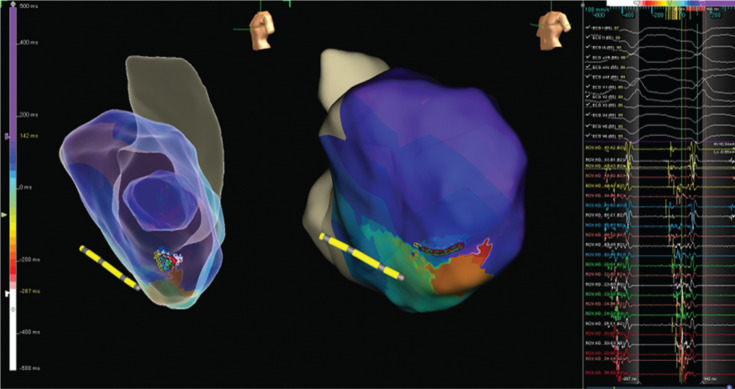
Nonsustained VT1 mapped with the Advisor™ HD Grid catheter and showing mid-diastolic potentials at the slow-conduction zone.

**Figure 8:**
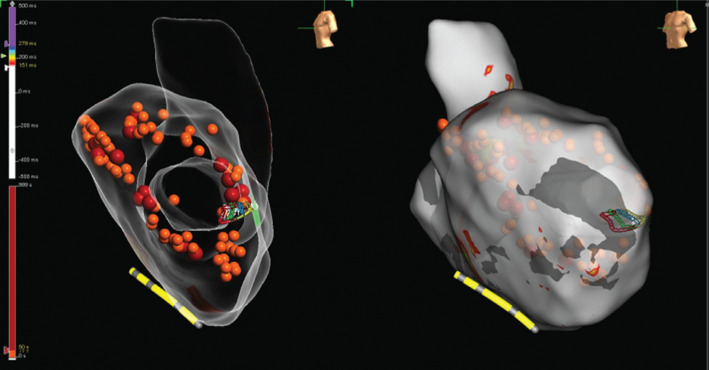
Activation mapping performed after ablation showing complete abolition of the late potentials.

**Video 1:** VT1 Propagation map: **A**: Left lateral and left anterior oblique views of VT propagation around the apical cannula. **B**: Color-coded activation map showing a slow conduction zone during VT located at the inferior aspect of the apical canula.
